# Ready-to-use food supplement, with or without arginine and citrulline, with daily chloroquine in Tanzanian children with sickle-cell disease: a double-blind, random order crossover trial

**DOI:** 10.1016/S2352-3026(18)30020-6

**Published:** 2018-03-13

**Authors:** Sharon E Cox, Elizabeth A Ellins, Alphonce I Marealle, Charles R Newton, Deogratias Soka, Philip Sasi, Gian Luca Di Tanna, William Johnson, Julie Makani, Andrew M Prentice, Julian P Halcox, Fenella J Kirkham

**Affiliations:** a Department of Population Health, London School of Hygiene & Tropical Medicine, London, UK; b Tropical Epidemiology Group, London School of Hygiene & Tropical Medicine, London, UK; c Medical Research Council Unit The Gambia, London School of Hygiene & Tropical Medicine, London, UK; d School of Tropical Medicine and Global Health, Nagasaki University, Nagasaki, Japan; e Muhimbili Wellcome Programme, School of Medicine, Muhimbili University of Health and Allied Sciences, Dar es Salaam, Tanzania; f Department of Clinical Pharmacy and Pharmacology, School of Pharmacy, Muhimbili University of Health and Allied Sciences, Dar es Salaam, Tanzania; g Department of Haematology, School of Medicine, Muhimbili University of Health and Allied Sciences, Dar es Salaam, Tanzania; h Department of Clinical Pharmacology, School of Medicine, Muhimbili University of Health and Allied Sciences, Dar es Salaam, Tanzania; i Institute of Life Sciences, Swansea University Medical School, Swansea University, Swansea, UK; j Department of Psychiatry, University of Oxford, Warneford Hospital, Oxford, UK; k Centre for Primary Care and Public Health, Queen Mary University of London, London, UK; l School of Sport, Exercise and Health Sciences, Loughborough University, Loughborough, Leicestershire, UK; m Developmental Neurosciences Programme, UCL Great Ormond Street Institute of Child Health, London, UK; n Clinical and Experimental Sciences Unit, Faculty of Medicine, University of Southampton, Southampton, UK; o University Child Health, University Hospital Southampton, Southampton, UK

## Abstract

**Background:**

Sickle-cell disease increases the risk of malnutrition. Low arginine and nitric oxide bioavailability are implicated in morbidity related to sickle-cell disease. Simple interventions are required, especially in low-income settings. We aimed to test the hypotheses that: (1) supplementary arginine, citrulline, and daily chloroquine increase bioavailable arginine and flow-mediated dilatation (FMD; maximal diameter change; FMD_max_%), a measure of nitric oxide-dependent endothelial function; and (2) protein energy supplementation in the form of ready-to-use supplementary food (RUSF) improves the height-for-age and body-mass index-for-age *Z*-scores in children with sickle-cell disease.

**Methods:**

We performed a double-blind, random order crossover trial with two 4-month intervention periods (each followed by 4-month washout periods) in Muhimbili National Hospital in Dar-es-Salaam, Tanzania. We enrolled 119 children from the Muhimbili Sickle Cohort who were aged 8–12 years, naive to hydroxyurea, and had documented HbSS phenotype. Two formulations of RUSF (providing 500 kcal/day) were tested: basic (RUSF-b), with which children also received weekly chloroquine (150 mg or 225 mg chloroquine base, dependent on bodyweight); and vascular (RUSF-v), which was fortified with arginine and citrulline (designed to achieve mean intakes of 0·2 g/kg per day of arginine and 0·1 g/kg per day of citrulline), and with which children received daily chloroquine (maximum 3 mg chloroquine base/kg per day). Children were randomly allocated to receive either RUSF-b first or RUSF-v first and, after a washout period, were then given the other treatment. The primary outcomes in comparing the two RUSF formulations were mean plasma arginine, arginine to ornithine ratio, and arginine to asymmetric dimethylarginine ratio, and mean FMD_max_%. The primary outcomes of the combined effect of both RUSF interventions were mean height-for-age *Z*-score and body-mass index-for-age *Z*-score. Analyses were done on the eligible intention-to-treat population. This trial is registered with ClinicalTrials.gov, number NCT01718054; and with ISRCTN74331412.

**Findings:**

Between Aug 9, 2012, and Feb 26, 2014, 145 children were randomised (71 children to RUSF-v first and 74 children to RUSF-b first) and 119 children were treated, of whom 114 children yielded complete data for all reported endpoints. The ratio of arginine to ornithine (mean of individual differences −8·67%, 95% CI −19·55 to 2·20; p=0·12) and the mean FMD_max_% (1·00, −0·47 to 2·47; p=0·18) did not significantly differ between the RUSF-b and RUSF-v treatments. However, the arginine to asymmetric dimethylarginine ratio was significantly increased by RUSF-v compared with RUSF-b (56·26%, 31·13 to 81·38; p<0·0001). In planned analyses that used mixed effects models to estimate the effect of each intervention compared with the participants at baseline or during washout periods, the arginine to asymmetric dimethylarginine ratio increased following both RUSF-v treatment (86%; p<0·0001) and RUSF-b treatment (40%; p<0·0001). However, FMD_max_% was higher after treatment with RUSF-v (0·92; p<0·0001) but not RUSF-b (0·39; p=0·22). Following either intervention (RUSF-b and RUSF-v, pooled) body-mass index-for-age *Z*-score (0·091; p=0·001) and height-for-age *Z*-score (0·013; p=0·081) increased compared with baseline and washout timepoints. In 83 participants in the treated population, there were 71 adverse events during the intervention, of which 21 (30%) were serious, and 81 adverse events during the washout periods, of which 26 (32%) were serious (p=0·31), including one patient who died in the second washout period.

**Interpretation:**

RUSF providing 500 kcal/day results in small weight gains in children with sickle-cell disease. However, even without arginine and citrulline fortification, RUSF seems to ameliorate arginine dysregulation and might improve endothelial function. Long-term studies are required to assess whether these physiological effects translate to improved clinical outcomes and better growth and development in patients with sickle-cell disease.

**Funding:**

Wellcome Trust.

**Research in context**

**Evidence before this study**

Before completion of the protocol, we searched ClinicalTrials.gov and the ISRCTN.gov clinical trials registries for unpublished randomised trials using the search term “sickle” within conditions; “arginine” or “citrulline” or “chloroquine” within interventions; and “energy” or “protein” or “food” or “supplement” combined with “sickle” within conditions. Similar searches were done in PubMed and were restricted to papers in English that were published before September, 2011. No previous randomised trials of a protein energy intervention in humans with sickle-cell disease have ever been registered or reported.

**Added value of this study**

To our knowledge, this trial is one of the first reported intervention trials for people living with sickle-cell disease in Africa, where most affected people reside. RUSF with arginine and citrulline fortification and daily chloroquine did not significantly increase plasma global arginine bioavailability ratio or flow-mediated dilatation (as a measure of endothelial function) compared with unfortified RUSF and weekly chloroquine. However, in planned analyses of the effects of each intervention and both combined, we observed significantly improved global arginine bioavailability ratio, arginine to asymmetric dimethylated arginine ratios, improved measures of endothelial function, and small improvements in height-for-age and body-mass index-for-age *Z*-scores after both interventions compared with baseline and washout measurements. No effect on inflammation was observed, suggesting a weak effect of the daily anti-inflammatory dose of chloroquine.

**Implications of all the available evidence**

Protein energy malnutrition, along with associated micronutrient deficiencies, might underlie some of the metabolic and physiological irregularities observed in sickle-cell disease, including arginine dysregulation and reduced endothelial function. Treatment and prevention of protein-energy malnutrition warrants much higher clinical priority.

## Introduction

About 6 million people live with sickle-cell disease in Africa.^1^ As survival increases, the number of people requiring chronic care will increase while the costs and complexity of follow-up interventions limit implementation; these include prophylactic blood transfusions and hydroxyurea therapy to the maximum tolerated dose.

Reduced growth is frequently observed in children with sickle-cell disease.^2^, ^3^ Energy and nutrient supplies are reduced in sickle-cell disease, resulting from decreased dietary intake, increased metabolic rate, increased nutrient degradation, and impaired metabolic pathways.^4^, ^5^ The possibility that the complications of sickle-cell disease might be ameliorated by appropriate dietary supplementation of protein, energy, and micronutrients has received little attention.

Vasculopathy, an important cause of morbidity and mortality in sickle-cell disease,^6^ is associated with endothelial dysfunction. This dysfunction includes increased endothelial cell activation and adhesion, abnormal tone, responsiveness, and vessel architecture,^7^ which can be disrupted by intravascular haemolysis, increased oxidative stress, and decreased nitric oxide bioavailability. Arginase is released from ruptured erythrocytes, platelets, and the liver.^8^ High concentrations of arginase reduce plasma arginine,^9^ the sole substrate of endothelial nitric oxide synthase, thereby decreasing nitric oxide production. Ornithine is the degradation product of arginase and is a competitive inhibitor of arginine uptake by endothelial cells, thus the arginine to ornithine ratio could be crucial for endothelial nitric oxide synthase activity. In patients with sickle-cell disease, low plasma arginine is common,^10^, ^11^ and is further decreased during vaso-occlusive episodes and acute chest syndrome.^12^ Low arginine to ornithine ratios and high asymmetric dimethylated arginine (ADMA), an endogenous inhibitor of endothelial nitric oxide synthase, are associated with increased pulmonary artery pressure and death.^9^, ^13^, ^14^ Reduced nitric oxide bioavailability is implicated in the pathophysiology of several disorders involving endothelial function, including sickle-cell disease, hypertension, and chronic kidney disease.^15^, ^16^, ^17^ Improving arginine concentrations might improve outcomes in these disorders. In adults with sickle-cell disease, oral arginine reduced pulmonary artery pressure by 15·2%,^18^ and children with acute pain who were randomly allocated to receive intravenous arginine required less analgesia than did children receiving placebo.^19^ The effects of longer-term supplementation of arginine on endothelial function in sickle-cell disease or on clinically important endpoints such as pain is unknown.

Flow-mediated dilatation (FMD) is a non-invasive ultrasound-based technique for assessing endothelial function by measuring the vasodilator response of the brachial artery to increased blood flow, which is dependent on local nitric oxide bioavailability and is a validated marker of vascular endothelial function and risk of arterial disease development.^20^ Alterations to the vasodilator responses enable the exploration of mechanisms involved in the initiation and progression of pre-clinical vascular disease in children^21^ and the evaluation of interventions in clinical trials.^22^ Endothelial function is abnormal in adults^23^, ^24^ and children^25^ with sickle-cell disease, and can improve after blood transfusion.^26^

We investigated whether arginine and citrulline fortification of a ready-to-use-supplementary food (RUSF), accompanied by an anti-inflammatory dose of chloroquine (a potential competitive inhibitor of arginase),^27^ increases bioavailable arginine and improves nitric oxide-mediated endothelial function, and tested whether RUSF improves nutritional status. The trial was designed to simultaneously address two separate questions: (1) the effect of arginine-fortified and citrulline-fortified RUSF on bioavailable arginine concentrations and endothelial function; and (2) the combined effect of RUSF formulation with and without these supplements on nutritional status.

## Methods

### Study design and participants

We did a double-blind, random order crossover trial of two RUSF formulations in children with sickle-cell disease: a basic formulation with weekly chloroquine (RUSF-b) or a vascular formulation (RUSF-v), which was fortified with arginine and citrulline and daily chloroquine. The trial was done in Muhimbili National Hospital in Dar es Salaam, Tanzania.

Eligible participants were children previously enrolled in the Muhimbili Sickle Cohort, for which the methods of recruitment and survival have been previously described.^1^ Children were enrolled between Aug 9, 2012, and Dec 3, 2012. All participants had been prescribed folate supplementation and advised to use insecticide-treated bednets. Chloroquine was not available before the start of this trial, since it was banned for treatment of malaria, and no other malaria prophylaxis was being implemented at the time of this trial.

The children lived in urban Dar-es-Salaam, had documented HbSS (determined by high-performance liquid chromatography [Variant II; Bio-Rad Laboratories, Hercules, CA, USA]), were aged 8–11 years at enrolment, and were naive to hydroxyurea therapy. Exclusion criteria and recruitment procedures are detailed in the appendix (pp 1–2). The key exclusion criteria were being overweight, low visual acuity, substantial renal or hepatic dysfunction (as indicated by abnormal clinical chemistry), and other serious comorbidities (such as receiving drugs known to interact with chloroquine).

The study was approved in Tanzania (National Institute of Medical Research and Muhimbili University of Health & Allied Sciences) and the UK (London School of Hygiene & Tropical Medicine). Parents or guardians provided written informed consent and children gave documented assent. The trial protocol is available online.

### Randomisation and masking

Patients were randomly assigned to receive either RUSF-b first, followed by RUSF-v, or RUSF-v first, followed by RUSF-b. The RUSF manufacturer generated the study participant identification codes in advance. Eligible participants were allocated a study identification code in the order that they were enrolled for laboratory screening for presence of abnormal liver or kidney function by the clinical research assistants in the study clinic. Participants were given an identification code and randomly assigned one of four treatment codes for each of the two treatment periods (indicated by different shapes printed on each, otherwise identical, RUSF packet) in blocks of 12 by use of the random function in Microsoft Excel. The same allocation code was used for the chloroquine, which was provided as a Monday to Saturday dosing bottle and Sunday dosing bottle that contained either base syrup alone or the appropriate concentration of chloroquine, as per the allocation code for each intervention period (appendix p 3). The list of identification numbers was linked to one of the four shape codes in another file accessed only by the field workers who delivered the intervention, but who were not involved in the outcome assessments at the clinic visits. The allocation code was known only to the RUSF manufacturer, the data safety monitoring board, and the study pharmacist. Investigators, clinical research assistants, field workers, care providers, and participants were blinded to the allocation code until final data analysis.

### Procedures

The randomly allocated interventions were administered for 4 months, followed by a 4-month washout; the other intervention was then administered for 4 months, followed by a second 4-month washout (appendix p 1). We used this study design: (1) as a standard analysis of the two treatments, allocated in a random order, to compare RUSF-v with RUSF-b treatment; and (2) to assess changes in endpoints after each treatment compared with baseline and washout timepoints and, where no evidence of differential effects were observed or expected, to calculate the average effect of both treatments combined. This approach effectively treats the study as a non-randomised, before-and-after intervention study within the same participants.

Both interventions consisted of twice-daily administration of RUSF, which was manufactured by Nutriset, (Malaunay, France), and was based on their supplementary PlumpyNut, which is used in the treatment of moderate malnutrition.^28^ This RUSF was comprehensively fortified with vitamins and minerals at approximately the recommended daily allowance (except for folate [1 mg/day] and iron [no fortification]) and provided 500 kcal/day (appendix p 3). RUSF-v was also fortified with 5·0 g (for participants with bodyweights of <25 kg) or 7·5 g (for participants with bodyweights of ≥25 kg) of L-arginine ketoglutarate and 2·5 g (bodyweights <25 kg) or 3·75 g (bodyweights ≥25 kg) of L-citrulline, to achieve mean intakes of 0·2 g/kg per day of L-arginine and 0·1 g/kg per day of L-citrulline and maximum intakes of 0·33 g/kg per day of L-arginine and of 0·165 g/kg per day of L-citrulline (appendix p 4). RUSF-v was administered with daily chloroquine syrup (Wallace Manufacturing Chemicals, Abingdon, UK) at either 50 mg (bodyweights <25 kg) or 75 mg (bodyweights ≥25 kg) chloroquine base to achieve a maximum dose of 3 mg chloroquine base/kg per day. RUSF-b was administered with weekly chloroquine at 150 mg (bodyweights <25 kg) or 225 mg (bodyweights ≥25 kg) chloroquine base per week. The RUSF packs and chloroquine were delivered fortnightly by study field workers who collected used chloroquine bottles and unused RUSF packs and assessed compliance (appendix p 4).

Participants attended the study clinic at enrolment (baseline) and at the end of each intervention and washout period (clinic visits 0–4; where baseline=0, end of intervention 1=1, end of washout 1=2, end of intervention 2=3, and end of washout 2=4). During these visits, a brief clinical examination was done and, if the participant was determined to be clinically well, the outcome measures were assessed. Participants who were found to be unwell were booked for another study clinic visit the following week. Participants continued to receive the intervention or remain in washout until they were judged to be clinically well at a clinic visit.

At each clinic visit, a 5 mL blood sample was collected. Aminoacid concentrations were measured by ion-exchange elution (Biochrom-30; Biochrom, Cambridge, UK) of frozen lithium heparin plasma, which was frozen within 2 h of collection at clinic visits 0, 1, and 3 (appendix p 5). Complete blood count and haemoglobin concentrations were measured at all clinic visits from ethylenediaminetetraacetic acid–whole blood (Sysmex XT2000i; Sysmex, Kobe, Japan; or ABX Pentra 60; Horiba, Kyoto, Japan). The concentrations of lactate dehydrogenase, total and direct bilirubin, aspartate transaminase, alkaline phosphatase, and creatinine were measured from fresh serum, taken at clinic visits 0–3, by use of an automated analyser (Cobas INTEGRA 400 plus; Roche, Basel, Switzerland). Visual acuity was assessed at all clinic visits using a modified Snellen chart.^29^

Nitric oxide-dependent endothelial function was determined by FMD. FMD was assessed by one of two trained operators using high resolution ultrasound (Ultrasonix, Vancouver, Canada), in accordance with a standard protocol^21^ and verified by one of the investigators (EAE; appendix pp 5–6). Briefly, dilatation of the right brachial artery during reactive hyperaemia (maximal diameter change, % [FMD_max_%]) was assessed after a 5-min cuff occlusion of the forearm. Changes in blood flow were also measured, and reactive hyperaemia was expressed as absolute change between baseline and peak velocity time integral (VTI). Endothelium-independent responses to 5 μg sublingual glyceryl trinitrate were also assessed and expressed as percentage change in diameter.

Height and weight plus body composition (measured with a Tanita BC-418 Segmental Body Composition Analyser [Tanita, Tokyo, Japan]) were assessed by one of three trained clinical officers at each of the five clinic visits. Throughout the study, field workers administered short questionnaires on clinical complications, such as pain, when delivering the intervention at fortnightly home visits and by telephone in intervening weeks.

### Outcomes

The primary outcome for a hypothesised effect of RUSF-v was mean differences between the RUSF-b and RUSF-v treatment groups in mean plasma aminoacid concentrations (arginine, arginine to ornithine ratio, and arginine to ADMA ratio) and in FMD_max_%. This outcome was assessed in all patients at clinic visits 0, 1, and 3 for aminoacid concentrations and clinic visits 0, 1, 2, and 3 for FMD_max_%. The primary outcome for a hypothesised effect of both RUSF interventions combined was mean height and body-mass index (BMI) as *Z*-scores-for-age (by use of UK 1990 reference values in the zanthro program^30^ in Stata 14.1 [StataCorp, College Station, TX, USA]). This outcome was assessed at baseline and following both treatments and washouts in all patients.

The secondary outcomes were changes to mean haemoglobin concentration, haemolytic markers, C-reactive protein concentration, and number of reported painful episodes associated with RUSF treatments. Glomerular filtration rate was also a secondary outcome, but will be reported elsewhere. Visual acuity, serum liver and kidney function markers, and complete blood counts were safety monitoring outcomes.

### Statistical analysis

A planned sample size of 120 was estimated to have more than 95% power to detect a potentially clinically important effect size of a change in FMD_max_% of 1·25 units, equivalent to a 16% change in FMD_max_% between RUSF-v and RUSF-b^21^, ^31^ and a 20% difference in growth rates for height and weight in the RUSF intervention growth periods compared with the washout (appendix p 8).

Analysis was by intention to treat of the eligible study population and was done by use of Stata 14.1. Possible carry-over effects were assessed by non-paired *t* tests of the average of the observations after the two treatment periods (eg, average of value at time 1  plus value at time 3), and by treatment order (RUSF-v first or RUSF-v second;^32^ p values >0·1 accepted as no carry-over). The effect of RUSF-v compared with the effect of RUSF-b on outcomes within subjects, allowing for the presence of an order effect, was first assessed using non-paired *t* tests of the average difference between treatments (eg, average of values at time 3 [after the second treatment] minus values at time 1 [after the first treatment]) by the order in which RUSF-v was received.^32^ We then used mixed-effects general linear regression models, which account for repeated observations within individuals (measurement occasion at level one; individuals at level two), to estimate the effects of each intervention compared with the baseline and washout values, and the effect of both treatments combined. Linear combination of effect estimates was used to estimate the effect of both RUSFs combined (ie, [β_1_ + β_2_]/2). Details of the models used are included in the appendix p 7–8. Due to several primary outcomes, p values <0·01 were taken as only indicative of a possible difference and values not reaching <0·001 were interpreted with caution. p values between 0·001 and 0·01 were also interpreted with caution. No interim analyses were planned or done. The trial was overseen by a data safety monitoring committee. The trial is registered with ClinicalTrials.gov, number NCT01718054; and with ISRCTN74331412 (Jan 18, 2012).

### Role of the funding source

The funder of the study had no role in study design, data collection, data analysis, data interpretation, or writing of the report. All authors had full access to all the data in the study and had final responsibility for the decision to submit for publication.

## Results

**Figure 1:**
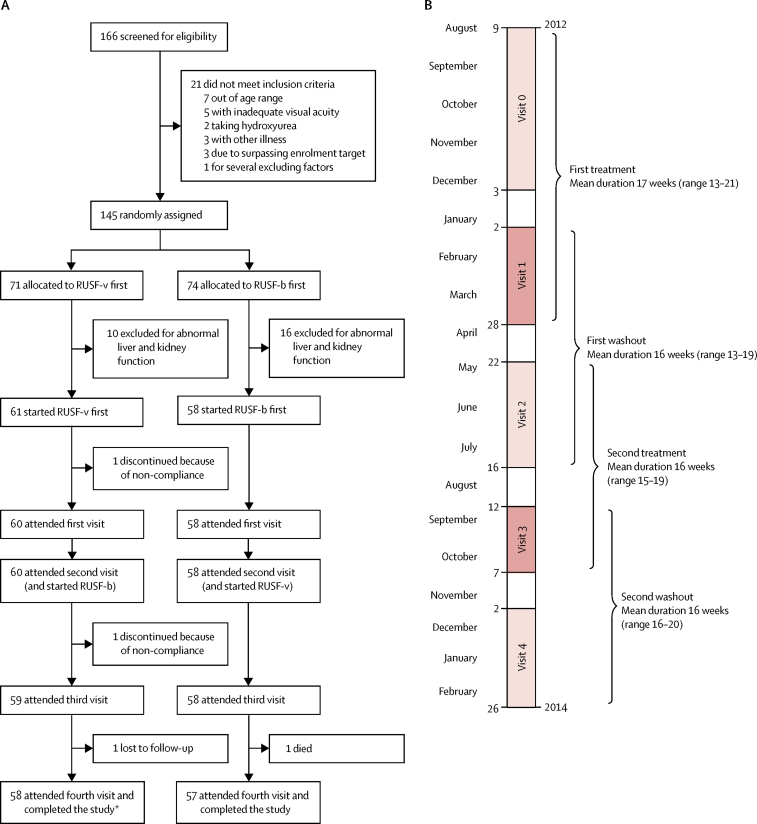
Trial profile (A) Trial profile. (B) Timeline. RUSF=ready-to-use supplementary food. RUSF-v=vascular treatment. RUSF-b=basic food. *One participant had missing endothelial data.

Between July 16, 2012, and Aug 3, 2012, 166 potentially eligible children were identified from the Muhimbili Sickle Cohort database. No families refused to participate. Screening and enrolment were completed between Aug 9, 2012, and Dec 3, 2012. 145 children were determined to be clinically eligible during screening and the baseline clinic visit (figure 1), and they were randomly assigned to treatment groups (71 children to RUSF-v first and 74 children to RUSF-b first). 26 children were later found to be ineligible because of abnormal kidney and liver function tests (as per the exclusion criteria; appendix p 1). 119 children were therefore initiated on the RUSF treatments (61 children in the RUSF-v first group and 58 children in the RUSF-b first group). 115 of these children completed the trial, attended all clinic visits, and had complete data (for aminoacid concentrations and anthropometry), and 114 also had complete endothelial function data. Participants with bodyweights of less than 25 kg and those with bodyweights of equal to or greater than 25 kg were evenly distributed between the two orders of treatment. At baseline (visit 0), 17 (28%) of 61 patients in the RUSF-v first group and 16 (28%) of 58 patients in the RUSF-b first group were at least 25 kg; at visit 3, the start of the second intervention, 31 (53%) of 59 patients in the RUSF-v first group and 26 (45%) of 58 patients in the RUSF-b first group were at least 25 kg.

Demographic, clinical, anthropometric, and laboratory values (including aminoacid concentrations) at baseline, both stratified by sex and combined, are reported in table 1. More boys (71 boys [60%] of 119 total patients) were recruited and were slightly older than girls. Variables that differed significantly by sex were haemoglobin concentration (higher in girls; p=0·017), fat-free body mass (lower in girls; p=0·002), and body fat percentage (greater in girls; p<0·0001); arginine to ornithine ratio (p=0·0076), global arginine bioavailability ratio (p=0·013), arginine to ADMA ratio (p=0·035), and resting brachial artery diameter (p=0·016) were also lower in girls than in boys. The proportion of girls weighing at least 25 kg at baseline (ten [21%] of 48), and therefore receiving the higher concentration of RUSF-v, was lower than in boys (23 [32%] of 71; p=0·17).

**Table 1:** Baseline characteristics

		**Total (n=119)**	**Male (n=71)**	**Female (n=48)**
**Age**				
Age, years		10·01 (1·25)	10·14 (1·20)	9·81 (1·30)
**Clinical observations**				
SpO_2,_%		98·97% (1·31)	98·82% (1·46)	99·21% (1·03)
Systolic blood pressure, mm Hg		102·82 (7·53)	103·55 (7·42)	101·75 (7·64)
Diastolic blood pressure, mm Hg		59·27 (7·58)	58·58 (7·09)	60·29 (8·21)
Palpable spleen, n (%)		18 (15%)	13 (18%)	5 (10%)
Jaundice, n (%)				
	None	0	0	0
	Mild	54 (45%)	31 (44%)	23 (48%)
	Moderate	52 (44%)	31 (44%)	21 (44%)
	Severe	13 (11%)	9 (13%)	4 (8%)
**Blood markers**				
Haemoglobin, g/dL*		7·49 (1·09)	7·29 (1·07)	7·78 (1·07)
White cell count, × 10^9^ cells per L*		13·44 (3·43)	13·59 (3·53)	13·23 (3·31)
Platelet count, × 10^9^ cells per L*		449·55 (175·35)	452·64 (168·85)	445·10 (186·01)
Median C-reactive protein (IQR), mg/L		3·30 (1·60–6·60)	3·60 (1·60–7·00)	2·70 (1·55–6·30)
Median lactate dehydrogenase (IQR), U/L		597 (497–677)	585 (481–685)	607 (516–659)
Median total bilirubin (IQR), mg/dL		36·4 (25·6–47·9)	34·0 (24·8–46·6)	40·9 (29·7–54·4)
Median non-conjugated bilirubin (IQR), mg/dL		10·6 (8·3–13·2)	10·4 (8·0–11·9)	11·2 (9·0–13·5)
Median creatinine (IQR), μmol/L		22·0 (18·0–26·5)	22·8 (18·1–26·9)	21·4 (18·0–24·9)
**Endothelial function**				
Flow-mediated dilatation, %		7·66% (3·37)	7·62% (3·54)	7·72% (3·12)
Resting brachial diameter, mm		2·61 (0·35)	2·67 (0·32)	2·52 (0·38)
Resting blood flow (VTI), m		0·20 (0·10)	0·20 (0·10)	0·20 (0·10)
Absolute reactive hyperaemia, m		0·69 (0·22)	0·72 (0·22)	0·66 (0·21)
**Endothelium-independent vasodilatation**				
Response to glyceryl trinitrate, %		4·15% (1·72)	4·22% (1·74)	4·03% (1·69)
**Aminoacid concentrations**				
Median arginine (IQR), μmol/L		54·40 (46·56–65·18)	55·62 (48·18–66·70)	51·79 (43·22–60·07)
Median citrulline (IQR), μmol/L		24·80 (21·28–28·50)	24·54 (20·58–29·08)	24·86 (21·62–28·23)
Median ornithine (IQR), μmol/L		52·80 (43·76–64·56)	51·78 (43·54–63·40)	55·55 (46·84–65·72)
Median arginine to ornithine ratio (IQR)		1·05 (0·88–1·28)	1·07 (0·94–1·34)	0·93 (0·74–1·15)
Median global arginine bioavailability ratio (IQR)		0·70 (0·58–0·84)	0·73 (0·62–0·88)	0·64 (0·53–0·76)
Median arginine to asymmetric dimethylarginine ratio (IQR)		53·27 (43·89–73·78)	55·37 (46·43–76·19)	51·15 (35·85–66·26)
**Anthropometric measurements**				
Height, cm		126·5 (7·2)	126·9 (6·7)	125·9 (8·0)
Weight, kg		22·9 (3·6)	23·2 (3·2)	22·5 (4·0)
Fat-free body mass, kg		18·59 (2·77)	19·22 (2·49)	17·66 (2·94)
Body fat, %		18·76% (3·22)	17·03% (2·67)	21·30% (2·08)
Weight ≥25 kg, n (%)		33 (28%)	23 (32%)	10 (21%)
Height-for-age *Z*-score		−2·09 (1·01)	−2·14 (0·84)	−2·01 (1·22)
Weight-for-age *Z*-score		−2·58 (1·12)	−2·65 (1·03)	−2·48 (1·25)
Body-mass index *Z*-score		−1·71 (0·88)	−1·65 (0·86)	−1·79 (0·90)

Data are mean (SD) unless otherwise indicated. VTI=velocity time intervals. SpO_2_=peripheral capillary oxygen saturation.

* Total N is 117 because two full blood count results (from male patients) were lost before data entry.

The mean compliance with RUSF (including coverage of supply and unused sachets) was 95%; 13 (11%) of 119 participants had a mean compliance of less than 90% and two (2%) of 119 had a compliance of less than 80% (both of whom were discontinued). There was no difference in mean compliance between RUSF-v and RUSF-b treatment groups (p=0·47). Seven chloroquine syrup doses were reported to be consumed in the previous 7 days for 90% of doses, which did not significantly differ between the treatments (p=0·84).

Geometric means and 95% CIs of aminoacid concentrations at baseline (time 0), at the first washout, and after treatment (visit 1 or 3) are shown in table 2. The effects of the treatments, individually and combined, compared with baseline and washout measurements are shown in table 3, and adjusted effects are shown in the appendix (pp 11–12). In an analysis that was not prespecified, we compared the unadjusted effects of order of treatment by unpaired *t* tests (table 4).

**Table 2:** Concentrations of aminoacids, haemoglobin, C-reactive protein, and haemolytic markers and measures of endothelial and non-endothelial dependent vascular function before and after RUSF treatment

	**Baseline**	**RUSF-v**	**RUSF-b**	**Washout 1**
**Aminoacid concentrations**				
Arginine, μmol/L	54·40 (51·71–57·22)	83·27 (75·13–92·29)	61·84 (58·57–65·29)	..
Citrulline, μmol/L	24·53 (23·41–25·70)	25·52 (22·91–28·41)	21·59 (20·68–22·53)	..
Ornithine, μmol/L	51·54 (47·85–55·52)	76·50 (69·61–84·08)	54·44 (51·59–57·44)	..
Asymmetric dimethylarginine, μmol/L	0·98 (0·91–1·06)	0·83 (0·79–0·88)	0·82 (0·76–0·88)	..
Arginine to ornithine ratio	1·06 (0·98–1·14)	1·09 (1·03–1·15)	1·14 (1·08–1·20)	..
Global arginine bioavailability ratio	0·70 (0·67–0·74)	0·81 (0·77–0·85)	0·80 (0·77–0·84)	..
Arginine to asymmetric dimethylarginine ratio	53·74 (49·54–58·29)	99·78 (89·93–110·70)	75·50 (69·19–82·38)	..
Endothelial function*				
Flow-mediated dilatation, %	7·66% (7·04–8·27)	8·60% (7·97–9·23)	8·10% (7·53–8·66)	7·70% (7·11–8·29)
Brachial artery diameter at rest, mm	2·61 (2·55–2·67)	2·64 (2·58–2·71)	2·70 (2·63–2·78)	2·63 (2·56–2·70)
Baseline blood flow (VTI), m	0·20 (0·18–0·22)	0·22 (0·20–0·24)	0·24 (0·22–0·25)	0·19 (0·18–0·21)
Absolute reactive hyperaemia, m	0·69 (0·65–0·73)	0·77 (0·73–0·81)	0·78 (0·74–0·81)	0·78 (0·75–0·82)
Endothelium-independent vasodilatation*				
Response to glyceryl trinitrate, %	4·22% (3·81–4·64)	4·41% (4·04–4·78)	4·51% (4·10–4·92)	4·77% (4·43–5·11)
**Blood markers**				
Haemoglobin, g/dL*	7·5 (7·3–7·7)	7·6 (7·4–7·8)	7·7 (7·6–7·9)	7·3 (7·1–7·4)
C-reactive protein, mg/L	3·35 (2·95–3·81)	3·84 (3·21–4·59)	3·94 (3·25–4·79)	3·51 (2·90–4·25)
Lactate dehydrogenase, IU/L	566 (548–585)	611 (576–649)	596 (561–633)	545 (519–573)
Total bilirubin, mg/dL	38·07 (35·44–40·89)	34·34 (30·00–39·32)	36·88 (32·80–41·48)	38·95 (34·93–43·43)
Non-conjugated bilirubin, mg/dL	27·03 (24·81–29·45)	24·53 (21·29–28·25)	24·93 (21·55–28·85)	27·94 (24·50–31·85)

Data are geometric mean (95% CI) and are natural log-transformed, unless otherwise indicated. RUSF=ready-to-use food supplement. v=vascular. b=basic.

* Mean (95% CI).

**Table 3:** Unadjusted estimates from multilevel models testing RUSF interventions compared with baseline and washout values (where available)

	**RUSF-v**		**RUSF-b**		**RUSF combined**	
	Coefficient (95% CI)	p value	Coefficient (95% CI)	p value	Coefficient (95% CI)	p value
**Aminoacids**						
Arginine, mmol/L	53·72 (39·09 to 68·20)	<0·001	13·88 (3·05 to 24·60)	0·008	..	..
Citrulline, mmol/L	4·08 (−4·87 to 13·88)	0·41	−11·93 (−19·84 to −3·29)	0·008	..	..
Ornithine, mmol/L	47·69 (33·64 to 64·87)	<0·001	5·13 (−4·87 to 17·35)	0·31	..	..
Asymmetric dimethylarginine, mmol/L	−9·51 (−24·42 to −4·877)	<0·001	−18·94 (−25·91 to −11·30)	<0·001	..	..
Arginine to ornithine ratio	3·05 (−3·92 to 10·51)	0·40	7·25 (0 to 15·02)	0·04	..	..
Global arginine bioavailability ratio	15·02 (9·42 to 20·92)	<0·001	15·02 (8·33 to 20·92)	<0·001	..	..
Arginine to asymmetric dimethylarginine ratio	85·89 (64·87 to 109·59)	<0·001	40·49 (24·60 to 58·40)	<0·001	..	..
**Endothelial function**						
Flow-mediated dilatation, %*	0·92 (0·30 to 1·54)	0·004	0·39 (−0·23 to 1·02)	0·22	0·66 (0·15 to 1·17)	0·011
Brachial artery diameter at rest, mm	0·03 (−0·01 to 0·07)	0·14	0·09 (0·05 to 0·13)	<0·001	0·06 (0·03 to 0·09)	<0·001
Baseline blood flow (VTI), m	0·024 (0·007 to 0·041)	0·007	0·043 (0·025 to 0·060)	<0·001	0·033 (0·019 to 0·047)	<0·001
Absolute reactive hyperaemia, m	0·03 (−0·01 to 0·07)	0·12	−0·04 (−0·01 to 0·08)	0·039	0·034 (0·004 to 0·065)	0·026
**Endothelium-independent vasodilatation**						
Response to glyceryl trinitrate, %	−0·04 (−0·44 to 0·37)	0·85	0·04 (−0·37 to 0·44)	0·86	0·00 (−0·33 to 0·33)	0·99
**Blood markers**						
Haemoglobin, g/dL	0·23 (0·12 to 0·34)	<0·001	0·35 (0·23 to 0·4)	<0·001	0·29 (0·20 to 0·37)	<0·001
C-reactive protein, mg/L	14·47 (−2·46 to 34·35)	0·098	17·39 (−0·09 to 37·94)	0·051	15·92 (1·684 to 32·15)	0·027
Lactate dehydrogenase, IU/L	7·70 (2·44 to 13·23)	0·004	5·10 (0·08 to 10·37)	0·046	6·39 (2·21 to 10·73)	0·002
Total bilirubin, mg/dL	−12·20 (−19·72 to −3·98)	0·004	−4·0 (−12·25 to 5·02)	0·37	−8·19 (−14·66 to −1·23)	0·022
Non-conjugated bilirubin, mg/dL	−11·25 (−19·38 to −2·30)	0·015	−8·82 (−17·06 to 0·23)	0·056	−10·0 (−16·75 to −2·78)	0·007
**Anthropometric measurements**						
Height-for-age Z-score	..	..	..	..	0·013 (−0·002 to 0·028)	0·081
Weight-for-age Z-score	..	..	..	..	0·070 (0·034 to 0·106)	<0·001
Body-mass index-for-age Z-score	..	..	..	..	0·091 (0·039 to 0·143)	0·001
Fat-free body mass	..	..	..	..	0·14 (−0·25 to 0·31)	0·094
Body fat percentage	..	..	..	..	0·27 (0·04 to 0·49)	0·022

Estimates were assessed by use of natural log-transformed concentrations of aminoacids and blood component concentrations, except for haemoglobin; for these variables, estimates are the exponential and equivalent percentage change. RUSF=ready-to-use food supplement.

* One extreme outlier value at baseline excluded.

**Table 4:** Comparison of effects of RUSF-v and RUSF-b, showing mean within individual differences by treatment order

		**Median (IQR) at time 1**	**Median (IQR) at time 3**	Mean within individual differences (SD)*	RUSF-v treatment effect (95% CI)*	p value*
**Aminoacids**						
Arginine, μmol/L						
	RUSF-v first	74·84 (58·13–99·35)	57·66 (51·14–81·12)	−30·80 (64·83)	−60·01 (−82·17 to −37·85)	<0·0001
	RUSF-b first	58·25 (51·88–68·24)	77·17 (57·34–106·52)	29·21 (55·76)	..	..
Citrulline, μmol/L						
	RUSF-v first	21·91 (18·10–28·69)	21·10 (18·82–25·18)	−18·45 (67·47)	−33·03 (−55·46 to −10·61)	0·0042
	RUSF-b first	21·90 (19·08–25·48)	22·56 (19·48–27·70)	14·59 (54·12)	..	..
Ornithine, μmol/L						
	RUSF-v first	64·80 (52·53–102·37)	49·38 (41·78–66·46)	−48·50 (69·92)	−72·13 (−94·74 to −49·53)	0·0001
	RUSF-b first	53·68 (47·16–63·78)	68·81 (55·74–98·26)	23·64 (52·05)	..	..
Asymmetric dimethylarginine, μmol/L						
	RUSF-v first	0·78 (0·67–0·98)	0·82 (0·68–1·06)	5·46 (57·89)	−3·74 (−22·14 to 14·65)	0·6874
	RUSF-b first	0·78 (0·66–0·88)	0·83 (0·72–0·96)	9·20 (40·99)	..	..
Arginine to ornithine ratio						
	RUSF-v first	1·15 (0·96–1·33)	1·17 (1·01–1·48)	6·52 (26·92)	8·67 (−2·20 to 19·55)	0·12
	RUSF-b first	1·05 (0·91–1·36)	1·10 (0·83–1·38)	−2·15 (32·28)	..	..
Global arginine bioavailability ratio						
	RUSF-v first	0·84 (0·73–0·97)	0·84 (0·75–1·02)	1·55 (23·12)	−0·72 (−10·19 to 8·74)	0·88
	RUSF-b first	0·76 (0·66–0·97)	0·78 (0·66–1·03)	2·28 (28·37)	..	..
Arginine to asymmetric dimethylarginine ratio						
	RUSF-v first	97·53 (71·48–142·14)	78·64 (55·77–95·37)	−36·26 (73·31)	−56·26 (−81·38 to −31·13)	<0·0001
	RUSF-b first	75·58 (57·26–101·16)	94·49 (67·98–127·58)	20·00 (63·47)	..	..
**Endothelial function**						
Flow-mediated dilatation, %						
	RUSF-v first	8·20 (3·1) N=56	7·88 (3·20) N=56	−0·32 (4·02)	−1·00 (−2·47 to 0·47)	0·1821
	RUSF-b first	8·31 (2·91) N=58	8·98 (3·76) N=58	0·67 (3·93)	..	..
Brachial artery diameter at rest, mm						
	RUSF-v first	2·66 (0·38) N=58	2·69 (0·42) N=58	0·03 (0·20)	0·10 (0·03 to 0·18)	0·0100
	RUSF-b first	2·72 (0·39) N=58	2·64 (0·35) N=58	−0·08 (0·23)	..	..
Baseline blood flow (VTI), m						
	RUSF-v first	0·24 (0·09) N=58	0·21 (0·08) N=58	−0·03 (0·10)	0·04 (0·00 to 0·07)	0·0460
	RUSF-b first	0·27 (0·09) N=58	0·20 (0·09) N=58	−0·07 (0·10)	..	..
Absolute reactive hyperaemia, m						
	RUSF-v first	0·76 (0·17) N=56	0·80 (0·21) N=56	0·04 (0·23)	0·03 (−0·06 to 0·11)	0·5454
	RUSF-b first	0·75 (0·19) N=58	0·77 (0·23) N=58	0·02 (0·22)	..	..
Response to glyceryl trinitrate, %						
	RUSF-v first	4·01 (1·71) N=56	4·62 (2·02) N=56	0·61 (2·52)	0·15 (−0·91 to 1·20)	0·7862
	RUSF-b first	4·40 (2·41) N=57	4·86 (2·26) N=57	0·46 (3·12)	..	..
**Blood markers**						
Haemoglobin, g/dL						
	RUSF-v first	7·67 (1·04) N=59	7·55 (0 .96) N=59	−0·12 (0·64)	0·23 (−0·03 to 0·49)	0·0777
	RUSF-b first	7·94 (1·01) N=58	7·58 (1·09) N=58	−0·36 (0·77)	..	..
C-reactive protein, mg/L						
	RUSF-v first	2·90 (1·55–6·30)	3·60 (2·10–7·10)	118·91 (97·72)	−14·77 (−57·51 to 27·96)	0·94
	RUSF-b first	3·80 (1·80–8·20)	4·40 (1·80–8·50)	133·68 (107·48)	..	..
Lactate dehydrogenase, IU/L						
	RUSF-v first	628 (500–730)	587 (479–705)	643·77 (33·13)	7·31 (−5·13 to 19·76)	0·51
	RUSF-b first	589 (500–690)	617 (509–708)	636·46 (25·66)	..	..
Total bilirubin, mg/dL						..
	RUSF-v first	35·85 (20·60–48·10)	39·60 (28·30–58·70)	359·44 (49·72)	10·88 (−11·68 to 33·46)	0·39
	RUSF-b first	32·80 (22·20–44·50)	32·20 (26·20–56·10)	348·55 (58·63)	..	..
Non-conjugated bilirubin, mg/dL						
	RUSF-v first	23·19 (11·50–34·70)	28·20 (16·90–46·80)	318·02 (61·46)	9·10 (−18·79 to 37·00)	0·84
	RUSF-b first	21·30 (15·03–31·20)	20·55 (15·30–40·70)	308·92 (72·46)	..	..

RUSF=ready-to-use food supplement.

* Non-normally distributed variables were transformed for *t* tests with y=100*ln(x); for these, estimated effects can be interpreted as a percentage difference in treatment effect on the original scale,^33^ and a negative value indicates a positive RUSF-v treatment effect.

At baseline, there was no effect of age, sex, BMI *Z*-score, or weight category on aminoacid concentrations. There was no evidence of an order or carry-over effect of RUSF-v (arginine, p=0·35; ornithine, p=0·72; ADMA, p=0·96).

The raw values of FMD_max_%, brachial artery diameter at rest, blood flow at rest (as measured by VTI), absolute reactive hyperaemia, and response to glyceryl trinitrate at baseline by sex are shown in table 1. Means and 95% CIs of these measurements at baseline (time 0), after RUSF interventions (time 1 or 3), and at first washout (time 2) are shown in table 2. Baseline associations between these measures and potential covariates are shown in the appendix (p 10). Values were similar at washout compared with baseline, with the exception of reactive hyperaemia, which had increased at the first washout. There was no evidence of an order effect or carry-over effect on FMD_max_% (p=0·22).

When we assessed aminoacid concentration after each RUSF intervention compared with baseline (table 3), we found that, following RUSF-v treatment, there were significant increases to arginine, ornithine, the global arginine bioavailability ratio, and the arginine to ADMA ratio, and a decrease to ADMA (all p<0·0001). After RUSF-b treatment, there was an increase in arginine (p=0·008) and a significant increase to the global arginine bioavailability ratio and the arginine to ADMA ratio, and a significant decrease in ADMA (all p<0·0001). The global arginine bioavailability ratio (arginine/ornithine + citrulline) increased to a similar extent after either treatment compared with baseline (15% increase; p<0·0001).

There was a greater decrease in ADMA after the RUSF-b intervention (18·9%) than after the RUSF-v intervention (9·5%; p<0·0001 for both treatments), but this difference was not significant (table 3). There was a greater increase in the arginine to ADMA ratio after RUSF-v treatment (86%) than RUSF-b treatment (40%), but this difference was not significant (table 3).

We found that, compared with RUSF-b, RUSF-v treatment significantly increased plasma arginine, ornithine, and the arginine to ADMA ratio and, to a lesser extent, citrulline. The increases to arginine and ornithine concentration were of similar magnitude within each group and, therefore, there was no difference to the arginine to ornithine ratio between RUSF-b and RUSF-v treatment groups.

When we assessed FMD_max_% after each RUSF intervention compared with the baseline and washout timepoints (table 3), FMD_max_% was increased following RUSF-v treatment (β coefficient 0·92; p=0·004). FMD_max_% was also significantly increased (1·19; p<0·0001) in the model adjusted for a priori covariates of age, sex, weight category, and other known important physiological determinants of the FMD_max_% response (heart rate, brachial artery diameter before cuff inflation, reactive hyperaemia,^34^ and C-reactive protein as a measure of inflammatory status;^35^ appendix pp 11–12). FMD_max_% was not increased following RUSF-b treatment, but there was an increase in the adjusted model (0·93, p=0·008). Neither treatment affected endothelium-independent vasodilator response to sublingual glyceryl trinitrate (table 3). FMD_max_% did not appear to differ between the RUSF treatments; however, the combined estimate of the average effect of both RUSF interventions (compared with baseline and washout timepoints) showed an increase in FMD_max_% of 0·66 (p=0·011; table 3) or 1·04 (p<0·0001) in the adjusted model (appendix p 11). Compared with RUSF-b there was no effect of RUSF-v on FMD_max_% (table 4).

**Figure 2:**
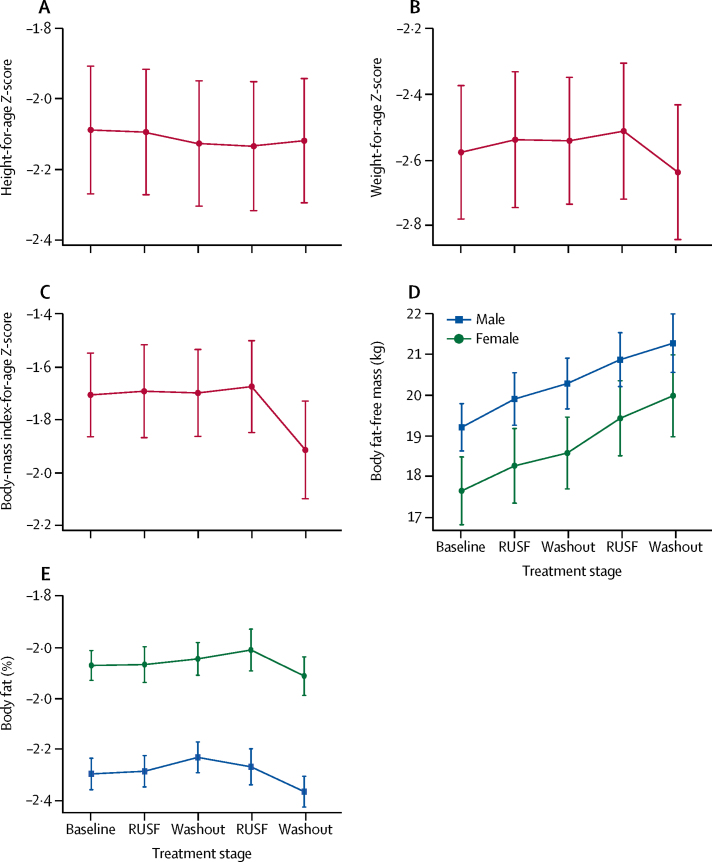
Anthropometry at each clinic visit Data are mean ± 95% CI. At each timepoint: (A) height-for-age *Z*-scores; (B) weight-for-age *Z*-scores; (C) body-mass index-for-age *Z*-scores; (D) body mass, stratified by sex; and (E) body fat, stratified by sex.

Mean BMI-for-age *Z*-score, height-for-age *Z*-score, weight-for-age *Z*-score, fat-free body mass, and body fat percentage at each clinic visit are shown in figure 2. Following either RUSF intervention (compared with the baseline or after either washout), the combined estimate of the average effect of both treatments indicated significant but very small increases in weight-for-age *Z*-score and BMI-for-age *Z*-score (table 3). In a preplanned analysis model that was adjusted for age, sex, and seasonality, the effects of treatment increased slightly and were significant (p<0·0001; appendix pp 11–12).

Resting blood flow and reactive hyperaemia following treatment did not differ between the RUSF-b and RUSF-v interventions (table 4). The combined estimate of the average effect of both RUSF interventions (compared with the baseline and washout timepoints) showed a significant increase in VTI (0·0033 m; p<0·0001) and a non-significant increase in reactive hyperaemia (0·0034 m; p=0·026; table 3).

Haemoglobin concentration was slightly but significantly increased following the RUSF treatments (compared with the baseline and washout timepoints); the combined estimate for the average effect of both treatments was an increase of 0·29 g/dL (p<0·0001). C-reactive protein, lactate dehydrogenase, total and unconjugated bilirubin concentrations did not differ after either RUSF intervention (table 3). However, in both treatments relative to baseline, we observed non-significant increases to lactate dehydrogenase and decreases to total and unconjugated bilirubin. In models adjusted for age, sex, and weight category, the combined estimate for the average effect of both interventions we also found a non-significant decrease in non-conjugated bilirubin with both treatments (p=0·001; appendix pp 11–12).

152 medical adverse events occurred in 83 participants, including 47 severe adverse events. Only 11 (7%) of 152 adverse events were seen at Muhimbili National Hospital. The one death was associated with previous vaso-occlusive pain and seizures in the second washout period. Similar numbers of adverse events occurred in the intervention (71 events; 21 events were serious) and washout periods (81 events; 26 events were serious; p=0·31; table 5). Vaso-occlusive coded adverse events were more frequent in intervention periods (29 events) compared with washout times (17 events; p=0·065), with no difference between treatments (table 5). There were no children with visual acuity less than 6/9 in either eye at any clinic visit.

**Table 5:** Causes and grade of adverse events by treatment periods

	**Intervention (n=71)**			**Washout (n=81)**			**Total (n=152)**
	RUSF-v	RUSF-b	Total	Washout 1	Washout 2	Total	
Abscesses	0	0	0	1 moderate	0	1	1
Abdominal pain	0	1 moderate	1	2 moderate	0	2	3
Anaemia	0	0	0	1 moderate	0	1	1
Acid reflux	1 severe	0	1	0	0	0	1
Body weakness	0	0	0	1 mild	0	1	1
Chest tightness	1 severe	0	1	0	0	0	1
Chickenpox	1 moderate	0	1	1 moderate	0	1	2
Diarrhoea	1 moderate	0	1	0	3 moderate	3	4
Epigastric pain	0	0	0	1 severe	0	1	1
Fever with source	0	1 severe	1	1 mild	0	1	2
Fever without source	4 moderate	1 moderate	5	3 (2 moderate, 1 severe)	0	3	8
Headache	0	1 severe	1	0	0	0	1
Malaria	8 (1 mild, 2 moderate, 5 severe)	10 (1 mild, 8 moderate, 1 severe)	18	26 (2 mild, 16 moderate, 8 severe)	11 (9 moderate, 2 severe)	37	55
Measles	0	0	0	0	1 moderate	1	1
Muscle spasms	1 mild	0	1	0	0	0	1
Nosebleeds	0	1 moderate	1	0	0	0	1
Pulmonary tuberculosis	0	0	0	1 moderate	0	1	1
Stroke	1 moderate	0	1	0	0	0	1
Tonsilitis	2 moderate	0	2	1 mild	2 (1 moderate, 1 severe)	3	5
Upper respiratory tract infections	2 moderate	4 (1 mild, 3 moderate)	6	3 (1 mild, 1 moderate, 1 severe)	0	3	9
Urinary tract infections	0	0	0	3 (1 moderate, 2 severe)	1 mild	4	4
Vaso-occlusive pain	14 (2 mild, 5 moderate, 7 severe)	15 (1 mild, 10 moderate, 4 severe)	29	12 (1 mild, 3 moderate, 7 severe, 1 very severe)	5 (1 mild, 3 moderate, 1 severe)	17	46
Vomiting	1 mild	0	1	0	0	0	1
Wounds	0	0	0	0	1 severe	1	1
Total	37	34	71	57	24	81	152

RUSF=ready-to-use food supplement.

Painful episodes (which were managed at home and recorded during weekly home visits or telephone calls) were also more frequent in the intervention periods (128 [5%] of 2555 assessments) compared with washout periods (92 [3%] of 3174 assessments; appendix p 13).

The number of adverse events related to laboratory findings at study visits increased after intervention periods compared with after the washout periods: the number of tests out of normal clinical range for aspartate transaminase was 49 (21%) of 234 (intervention) compared with five (2%) of 233 (washout); for alkaline phosphatase, 11 (5%) of 234 tests were out of normal range (intervention) compared with 0 of 233 tests (washout); and, for total bilirubin, ten (5%) of 234 tests were out of normal clinical range (intervention) compared with six (3%) of 233 tests (washout; appendix pp 13–14). However, these findings were mostly only of mild grade. Notably, there were fewer decreases in haemoglobin concentration after intervention periods (15 [6%] of 233 tests) than after washout (35 [15%] of 233 tests). There was no indication of any differences between the RUSF treatments with regard to their effects on haemoglobin concentration (table 4).

## Discussion

RUSF formulations, both with and without arginine and citrulline fortification and daily chloroquine, improve bioavailable arginine and might improve endothelium-dependent vasodilator function in Tanzanian children with sickle-cell disease.

Arginine and citrulline fortification and daily chloroquine in the RUSF-v intervention improved the concentration of bioavailable arginine (assessed by global arginine bioavailability ratio and arginine to ADMA ratio) and unexpectedly, the unfortified RUSF with weekly, low-dose chloroquine similarly increased global arginine bioavailability ratio and, to a lesser extent, the arginine to ADMA ratio in the patients. The effect of the RUSF-b intervention on the arginine to ADMA ratio appeared to be driven by a larger reduction in ADMA compared with baseline after RUSF-b treatment than after RUSF-v treatment, which, unsurprisingly, had a greater effect on arginine concentrations. Given evidence on the risk of cardiovascular outcomes in populations without sickle-cell disease, it seems likely that ADMA could be the most important determinant of vascular health.^36^ RUSF-v and RUSF-b groups did not significantly differ on measures of endothelial function. Effects of the RUSF interventions on anthropometric outcomes were significant but minimal. Both interventions appear safe, were well tolerated, and had good compliance.

The effect of oral L-arginine supplementation on vascular endpoints in sickle-cell disease adults is controversial. To our knowledge, this is the first trial of non-acute supplementation in children with sickle-cell disease. We found evidence to suggest a beneficial effect of both RUSF interventions on FMD_max_%, with no effect on response to glyceryl trinitrate including in analyses adjusted for factors that might otherwise affect underlying vasomotor responses such as vessel size, heart rate, and inflammatory status. These findings suggest that this vascular effect is not a consequence of a change in smooth muscle function, but is more likely to be endothelium-dependent. Although this improvement was unexpected for RUSF-b, the larger reduction in ADMA following RUSF-b than following RUSF-v could underlie this effect, and suggests that additional protein might improve dysregulated arginine metabolism in sickle-cell anaemia.^9^ Additionally, although not significant, RUSF-b might have had a greater effect on resting blood flow (VTI), which could suggest improved resting microvascular dilator tone associated with this treatment. Furthermore, the increase in FMD_max_% remained apparent after adjustment of relevant variables, including for reactive hyperaemia, suggesting that enhanced microvascular reactivity was also not responsible for the observed improvement in endothelium-dependent vasodilatation.

The effects of these interventions on BMI-for-age and height-for-age *Z*-scores were small, at least over this time period. Therefore, it would appear that the amount or duration of nutritional support, or both, were insufficient to greatly increase weight gain or to support linear growth. There was a marked drop in weight-for-age *Z*-scores and BMI-for-age *Z*-scores in the second washout period; body composition data suggested that this change was due to a decrease in fat mass while height-for-age *Z*-score increased. These findings suggest that resources were directed to linear growth in the second intervention at the cost of fat mass. An alternative interpretation would be an independent effect of season during the second washout period, although the fewer adverse events recorded in the second washout period makes this seem less likely.

Notably, there is only one published protein-energy supplementation study^37^ in sickle-cell disease, which investigated three children who were growth-retarded and were fed naso-gastrically (two children) or orally (one child); this study reported remarkable improvements in growth and clinical course in the two children fed naso-gastrically. Studies^38^ in transgenic mice for sickle-cell disease show a higher protein requirement than control mice, with maximal growth and reduced end-organ damage on a diet with 35% energy from protein. Increased protein, particularly increased arginine, also increased lean body mass, bone mineral density, and grip strength in these mice.^39^

We observed a significant small increase in haemoglobin concentration after RUSF intervention, along with evidence of decreased concentrations of bilirubin markers of haemolysis but increased lactate dehydrogenase relative to baseline and washout. In light of some increases in liver function tests after RUSF, this finding suggests that lactate dehydrogenase concentration might have increased due to effects on the liver. An absence of effect of RUSF-v containing daily chloroquine on C-reactive protein concentration does not support an anti-inflammatory effect of chloroquine, for which it has been used in conditions such as juvenile arthritis at similar doses.^40^

RUSF appears to be safe and well tolerated in Tanzanian children with sickle-cell disease, albeit with a possible increase in the frequency of pain-related adverse events and pain managed at home. This finding contrasts with evidence from a clinical trial^19^ of oral or intravenous arginine supplementation, which significantly reduced narcotic pain relief during acute painful episodes that required hospital treatment. However, there was quite a large difference in the number of adverse events between the first and second washout period; this difference could suggest an effect of seasonality, which is a limitation of crossover study designs and before-and-after intervention studies. This crossover trial in patients with limited access to emergency health care and imaging also limited the feasibility of assessing clinically important endpoints other than growth, pain, and haemoglobin concentration. Although mortality is high in Tanzania,^1^ only one child died in this relatively short study. Future studies of nutritional supplementation in other settings could report hospital admissions, (eg, for acute chest syndrome, CNS complications, or pain), outpatient prescriptions for analgesia, echocardiography or cardiac MRI for evidence of pulmonary hypertension or diastolic dysfunction, as well as brain MRI for volume or tissue characteristics as clinically relevant endpoints. Finally, we acknowledge that most of the significant observations of this study result from the analysis of the non-randomised study design component with implications for the strength of evidence. However, this design allowed us to simultaneously assess effects on several endpoints in a smaller sample size with high power and to avoid the potential ethical issue of a placebo arm in a traditional parallel treatment arm trial.

This trial supports the use of protein energy supplementation in relatively malnourished children with sickle-cell disease to improve arginine availability, haemoglobin concentration and, possibly, vascular endothelial function. There were few additional benefits of an arginine and citrulline fortified so-called nutraceutical RUSF compared with a basic RUSF. Larger trials of longer duration and, possibly, increased protein-energy supply will be needed to determine the effects on endpoints of importance to patients, including growth, pubertal and cognitive development, hospital admissions, pain, vascular events, and exercise tolerance.
