# Supplementary material for: Ready-to-use food supplement, with or without arginine and citrulline, with daily chloroquine in Tanzanian children with sickle-cell disease: a double-blind, random order crossover trial
Source: Lancet Haematol. 2018 Mar 13;5(4):e147–60. doi: 10.1016/S2352-3026(18)30020-6 (PMC5871644; doi:10.1016/S2352-3026(18)30020-6)
Supplement: Supplementary appendix [file mmc1.pdf]

THE LANCET
Haematology

Supplementary appendix

This appendix formed part of the original submission and has been peer reviewed. We post it as supplied by the authors.

Supplement to: Cox S E , Ellins E A, Marealle A I, et al. Ready-to-use food supplement, with or without arginine and citrulline, with daily chloroquine in Tanzanian children with sickle-cell disease: a double-blind, random order crossover trial. *Lancet Haematol* 2018; published online March 13. http://dx.doi.org/10.1016/S2352-3026(18)30020-6.

Supplementary Appendix

A. Study design, patient Recruitment procedures and exclusion criteria

Supplementary Figure 1

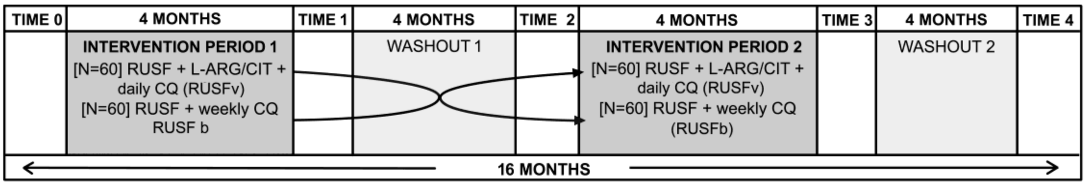

Parents or caregivers were contacted via telephone and visited at home by a study field worker to explain the nature of the study and invited to attend the V-FIT clinic for screening and enrolment if eligible.

Exclusion criteria were: (i) Body Mass Index (BMI) for age>95th percentile; (ii) low visual acuity (<6/9) in either eye assessed using a modified Snellen chart or previously diagnosed chronic eye disorder likely to suggest retinopathy or macular degeneration; (iii) significant renal or hepatic dysfunction as indicated by out of range values of any of serum creatinine, aspartate transaminase or alkaline phosphatase; (iv) diagnosed tuberculosis, or HIV stage III or above or receiving anti-retroviral therapy; (v) diagnosed or clinical signs of pulmonary hypertension or heart failure; (vi) diagnosed epilepsy, psoriasis or taking medications known to be contraindicated with chloroquine. Specifically, the following checklist items for inclusion and exclusion criteria were employed.

Blood samples for clinical chemistry analyses of kidney and liver function were drawn from children who were otherwise eligible and the baseline visit assessments proceeded with, including randomization, due to logistical restraints and the time required before clinical chemistry results were returned.

Does the subject meet the following INCLUSION criteria?

If any of these are NO the patient is NOT eligible for randomisation

		YES	NO
a.	Has informed consent been given and the form signed and witnessed if appropriate?	☐	☐
b.	Are the requirements for participating in the study understood?	☐	☐
c.	Is patient likely to stay in the study area for the duration of the study?	☐	☐
d.	Is the patient enrolled in the Muhimbili Sickle Cohort	☐	☐
e.	Is patient aged between 8.0 to 11.9 years of age today?	☐	☐
f..	Does the patient have HbSS or HbSB0 phenotype?	☐	☐

Does the patient have any of the following EXCLUSION criteria?

If any of these are YES the patient is NOT eligible for randomisation

		YES	NO
a.	Is the patient taking other significant long-term medications? [not including folic acid other micronutrient or nutritional supplements or penicillin prophylaxis]	☐	☐
b.	Is the patient taking any medications listed as interacting with chloroquine [see worksheet #2]	☐	☐
c.	Does the patient have stage III or IV HIV infection or taking anti-retrovirals?	☐	☐
d.	Does the patient have TB infection?	☐	☐
e.	Does the patient have any other clinically significant long-term illnesses other than SCD?	☐	☐
f.	Is the patient taking hydroxyurea?	☐	☐
g.	Has the patient had a blood transfusion in the last 30 days?	☐	☐
h.	Has the patient ever been diagnosed with cardiac problems or pulmonary hypertension?	☐	☐
i.	Has the patient ever been diagnosed with epilepsy?	☐	☐
j.	Has the patient ever been diagnosed with psoriasis?	☐	☐
k.	Has the patient ever been diagnosed with any chronic eye problems likely to indicate retinopathy or macular degeneration	☐	☐
l.	Does the patient have a serious eyesight deficiency [Snellen <6/9]?	☐	☐
m.	Does the patient have significant liver or kidney dysfunction<br>[Any of the following: serum creatinine, aspartate transaminase or alkaline phosphatase > 2 x upper limit of normal for SCD in this age range?] [see worksheet #3]	☐	☐

B. Interventions

The detailed composition of the RUSF interventions is shown in (Table S1).

Table S1. Detailed amino acid and micronutrient composition of RUSF intervention. Amount per day (2 packets)

	Daily dose	RUSF-b	RUSF-v (low, <25kg)	RUSF-v (high, ≥25kg)
Calories total	kcal	500	500	500
Proteins	g	13	13	13
Arginine	g	0	5	7.5
Citrulline	g	0	2.5	3.75
Vitamin A / Retinol	µg	600	600	600
Vitamin B1 / Thiamine	mg	1.1	1.1	1.1
Vitamin B2 / Riboflavin	mg	1	1	1
Vitamin B3 / Niacin	mg	16	16	16
Vitamin B5 / Pant. Acid	mg	5	5	5
Vitamin B6 / Pyridoxine	mg	1.2	1.2	1.2
Vitamin B8 / Biotin	µg	25	25	25
Vitamin B9 / Folate	µg	1000	1000	1000
Vitamin B12 / Cobalamin	µg	2.4	2.4	2.4
Vitamin C / Ascorbate	mg	45	45	45
Vitamin D / Calciferol	µg	15	15	15
Vitamin E / Tocopherol	mg	11	11	11
Vitamin K	µg	55	55	55
Phosphorus	mg	1250	1250	1250
Calcium	mg	1300	1300	1300
Potassium	mg	4.5	4.5	4.5
Iodine	µg	120	120	120
Iron	mg	0	0	0
Magnesium	mg	240	240	240
Selenium	µg	40	40	40
Copper	µg	700	700	700
Zinc	mg	11.2	11.2	11.2
Sodium	g	low	low	low

.

The weekly chloroquine dosage given with the RUSF-b consisted of 150mg or 225mg of chloroquine base, depending on low or high weight category (< or >=25 kg), contained within the "Sunday" syrup bottle, with daily doses of the base placebo syrup on the remaining days. The daily chloroquine dosage given with the RUSF-v consisted of 50 mg or 75mg by weight category with identical concentrations in the Sunday bottle and the Monday-Saturday bottle to blind participants to which chloroquine dosage they were receiving.

Dosage of L-arginine and L-citrulline

The final targeted dosages of the amino acids (0.2g/kg/d for L-arginine plus 0.1g/kg/d for L-citrulline) were chosen on the basis of three criteria: 1) safety; 2) evidence of efficacy; and 3) limitations on the amounts that can be incorporated into the RUSF, whist maintaining sufficiently high energy-density, taste and textural properties.

The observed safe level (OSL) for L-arginine is 20g/day in healthy adults (please see below). This is equivalent to 0.33g/kg/d assuming an average adult weight of 60kg. Doses of 0.1-0.2g/kg/day of L-arginine[1,2] & L- citrulline[3] have been utilized previously in SCD subjects with no adverse effects or toxicities reported and resulted in significant increases in plasma arginine concentrations. Three months' L-arginine supplementation (0.1-0.2g/kg/d) increased plasma arginine over time from baseline (50.1+/-17.0 µMol/L, N=8) to a near 100% increase at 12 weeks[1].

A lower dose of L-citrulline compared to L-arginine is proposed based on: i) doubled or greater AUC plasma arginine responses compared to L-arginine[4]; ii) relative expense of L-citrulline compared to L-arginine; and iii) less evidence available to determine observed safe levels.

Assessment of compliance

Using standard pro-forma, study field workers asked the parent or caregiver during alternating weekly home and telephone interviews how many RUSF packets and doses of the chloroquine syrup the study participant had received in the last 7 days. At home visits, they also recorded and collected any un-used RUSF sachets and remaining chloroquine syrup.

C. **Outcome measures.**

Amino acid concentrations: were measured in frozen lithium heparin plasma samples by ion-exchange elution (Biochrom-30, Biochrom, UK) at clinic visits 0, 1, and 3. Blood samples were processed and frozen within 20-120 minutes after blood collection. Prior to analysis samples were de-proteinized using 5% 5-Sulphosalicylic acid whereby 1 part of the plasma sample was mixed well with 1 part of 5% 5-Sulphosalicylic acid solution containing norleucine as an internal standard, centrifuged and the supernatant obtained for injection.

Endothelial function: flow mediated dilatation (FMD) was assessed in a temperature-controlled room after patients had rested for 10 minutes. The right brachial artery was imaged longitudinally using high resolution ultrasound (Ultrasonix, Vancouver, Canada). Once a good clear image was obtained the probe was fixed into position using a stereotactic clamp (ALOKA) and the image zoomed. A Doppler cursor was placed in the centre of the vessel to assess changes in blood flow. The image was triggered to the R-wave of the ECG so that end diastolic images were captured. Baseline brachial artery images and blood flow were recorded for 1 minute prior to the rapid inflation of the occlusion cuff positioned just below the medial epicondyle to 200mmHg. After 5 minutes the cuff was released and recording of the artery and blood flow continued for a further 3 minutes. Blood pressure readings were taken at the beginning and end of the studies. Images were saved on the ultrasound machine for later offline analysis. Diameter changes were assessed using edge detection software (Brachial Tools, Iowa City, Iowa). FMD was expressed as percentage change ([peak diameter – baseline diameter] / baseline diameter) x 100. Velocity time integral (VTI) of the Doppler trace was analysed using the flow analyser from Brachial tools. Baseline VTI, as a measure of blood flow at rest, was averaged from recordings during the first minute of assessment. Peak VTI was the maximum reading within the first 15 seconds following release of the occlusion cuff. Reactive hyperemia (RH) was expressed as absolute change (peak VTI – baseline VTI).

Endothelium-independent responses to 5µg sub-lingual glyceryl-trinitrate (GTN) were also assessed and expressed as percentage change ([peak diameter – baseline diameter] / baseline diameter) x 100. All measurements were made by one of two operators trained in the UK by the Halcox group, who also provided on-site training and quality control. The two operators underwent 6 weeks of training and

practice in the assessment and analysis of FMD, passed a pre-specified certification programme and demonstrated inter-operator reproducibility. Ongoing quality control involved checking by the senior research technician and investigator (E Ellins) that the quality of the images obtained was satisfactory, that the analysis was appropriate and the protocol adhered to.

D. Statistical analysis using mixed effects linear regression and sample size

We used mixed-effects general linear regression models, which account for repeated observations within individuals (measurement occasion at level one; individuals at level two), to estimate the effects of each intervention compared to baseline and washout values, and of the effect of both treatments combined. Wald tests were used to compare the estimates for each intervention (i.e., RUSF-v vs. RUSF-b). Post-estimation combination of linear effect estimates was used to estimate the effect of both RUSFs combined (i.e., (β_1 + β_2)/2).

Equation 1. Unadjusted models

$$y_{it} = \beta_{oi} + \beta_1 RUSFv_{it} + \beta_2 RUSFb_{\mathrm{it}} + \varepsilon_{it}$$

$$\beta_{oi} = \beta_o + u_{0i}$$

$$\varepsilon_{it} \sim N(0, \sigma_\varepsilon^2)$$

where y_{it} is the outcome in individual i at time point t, β_{oi} is the intercept for individual i comprised of a sample-average fixed effect (β_o) and an individual random effect (u_{0i}), and ε_{it} is the residual error. The u_{0i} $\sigma_{\mathrm{u}}^2$and $\mathrm{u}_{0\mathrm{j}}$ are assumed to be normally distributed with mean zero and variance σ_{uo}^2 and σ_ε^2, respectively. β_1 and β_2 are the intervention estimates; $RUSFv_{it}$ and $RUSFb_{it}$ were each coded as one at times points 1 and 3 (if that intervention had been given in the preceding period) and zero otherwise.

Models were expanded to include adjustment for covariates, selected based on theory, in order to improve precision of the intervention estimates.

Equation 2. Adjusted models

$$y_{it} = \beta_{oi} + \beta_1 RUSFv_{it} + \beta_2 RUSFb_{it} + \varepsilon_{it} + \beta_3 L2cov_i + \beta_4 L1covar_{it} + \beta_{5i} L1covar\_re_{it}$$

$$\beta_{oi} = \beta_o + u_{0i}$$
$$\beta_{5i} = \beta_5 + u_{5i}$$

$$\begin{pmatrix} u_{0i} \\ u_{5i} \end{pmatrix} \sim N\left\{\begin{pmatrix} 0 \\ 0 \end{pmatrix}, \begin{pmatrix} \sigma_{u0}^2 & \\ \sigma_{u05} & \sigma_{u5}^2 \end{pmatrix}\right\}$$

$$\varepsilon_{it} \sim N(0, \sigma_\varepsilon^2)$$

Where $L2cov_i$ is a level two covariate (e.g., sex), $L1covar_{it}$ is a level one covariate (e.g., age of measurement), and $L1covar\_re_{it}$ is a level one covariate with a random effect. The resulting variance-covariance matrix between the random effects was unstructured (i.e., σ terms were freely estimated with no constraints).

Sample size calculations at 90% power and 5% significance for two-sided test were estimated from the following formula:

$$N = 1 + Rx\left[\frac{1.96 + 1.28}{\frac{\Delta}{\sigma w}}\right]2$$

R = reliability of measurements = $\sigma_B^2/(\sigma_w^2+\sigma_B^2)$

Estimates of intra-individual variance = $\sigma_w^2 = SD^2/2$ and inter-individual variance = $\sigma_B^2 = SD^2/2$

The estimated sample sizes for 90% power and 5% significance were 28 for FMD % and 20 for nutritional status using estimates of intra- and inter-individual variance as outlined below.

1. FMD: The within-individual standard deviation (SD) for repeated measures over 3 months of FMD_{max} (%) using the same protocol, in 42 healthy British adults, is 1.04 units[5]. However, to be conservative we doubled this to 2.08. Mean FMD_{max} (%) in 18 British HbSS children with and without obstructive sleep apnoea, was 7.71 (SD) 6.27 (Kirkham, unpublished data) compared to 6.3 (SD) 5.4 in 31 British HIV+ children treated with protease inhibitor ARVs, who had significantly lower FMD_{max} (and greater variance) compared to HIV positive children not treated with protease inhibitor drugs[6]. Taking a conservative approach, we used the largest inter-individual SD of 6.27.

2. Growth rates: The mean within-individual growth rate in 181 Tanzanian HbSS children aged 8-11y at first measurement, with a minimum of 8 months' follow-up and 3 measurements is 0.377+/-0.283cm/month. The size of the SD compared to the mean (SD/mean = 0.75) is greater compared to Gambian children of the same age, collected under strict quality control conditions (SD 0.177/0.425 = 0.41). This is likely a function of greater inaccuracy in single measurements conducted in a busy clinic,

and genuine increased within-individual variance due to SCD status. In Gambians, an increase of 0.5 SD translates to a 20% increase in growth rate, which is a clinically significant effect likely to be observed in the duration of RUSF supplementation in this study. Between-individual variation (SD) in growth in the HbSS children was 0.234cm/month. Hence we based our calculations on an effect size of 0.5 x within-individual SD in Tanzanian HbSS children for height and weight growth rates .

E. Supplementary Results Tables

Table S2: Associations between measures of endothelial function at baseline using linear regression

Explanatory factor	FMD, %	Resting brachial diameter, mm	Resting VTI	Reactive hyperemia [RH]
FMD%				
Baseline brachial diameter, mm	-3.42 P=0.001			
Resting blood flow VTI	1.76 P=0.58	0.32 P=0.32		
Reactive hyperemia [RH]	1.47 P=0.31	0.33 P=0.82	0.22, P<0.0001	
GTN, %	0.31 P=0.090	-0.02 P=0.20	NA	NA

Table S3: Factors associated with measures of vascular function at baseline using linear regression, reporting the regression coefficient and p-value

Explanatory factor	FMD_{max} %*	Resting brachial diameter, mm	Resting VTI	Reactive hyperemia, [RH]	GTN, %*
Age, y	NS	0.05 P=0.091	NS	NS	NS
Male vs female	NS	0.16 P=0.016	NS	NS	NS
Weight category ≥25kg vs <25kg	NS	0.33 P<0.0001	NS	NS	NS
DBP at rest, mm Hg	NS	NS	-0.003 P=0.18	NS	NS
SBP at rest, mm Hg	NS	NS	NS	NS	NS
Heart rate at rest, bpm	0.06 P=0.077	-0.01 P=0.020	NS	-0.0065 P=0.003	0.04 P=0.44
Seasonality[1]	NS	NS	YES	NS	NS
Haemoglobin, g/dl	NS	NS	NS	NS	NS
CRP	NS	NS	NS	NS	NS
Plasma ARG z-score[2]	-0.15 P=0.61	-0.05 P=0.092	0.01 P=0.22	0.01 P=0.47	0.04 P=0.19
Plasma ARG:ORN z-score[2]	0.27 P=0.36	-0.03 P=0.29	-0.008 P=0.36	0.008 P=0.70	0.08 P=0.61
Plasma ARG:ADMA z-score[2]	-0.04 P=0.89	0.07 P=0.021	0.023 P=0.013	0.044 P=0.026	-0.004 P=0.91
BMI-z score	0.85 P=0.018	0.15 P<0.0001	NS	NS	NS
Height for age z-score	NS	0.09 P=0.005	NS	NS	NS
Lean body mass, kg	NS	0.06 P<0.0001	NS	NS	NS

** models adjusted for resting brachial diameter*
1 – Season is a continuous variable ranging from 0 to 1 [where 0 is the 1st January and 1 is the 31st December] and was modelled as the first two sets of Fourier terms (first set = Cosine(2πS) and Sine(2πS); second set = Cosine(4πS) and Sine(4πS))
2 – internal z-score, therefore coefficient represents effect associated with an increase of 1 standard deviation.

Table S4. Adjusted estimates from multilevel models testing the effects of RUSF interventions on natural log transformed amino acid concentrations, measures of endothelial function, nutritional status and body composition, hemoglobin and natural log transformed C-reactive protein and haemolytic markers.

	RUSF-v		RUSF-b		RUSF combined	
**Amino acids**	**Adj Coefficient (% change) [95% CI]**	**P-value**	**Adj Coefficient (% change) [95% CI]**	**P-value**		
Arginine, μmol/L	54.64 [39.0/72.07][a]	<0.0001	14.72 [3.02 – 27.75] [a]	0.012	na	na
Citrulline, μmol/L	5.67 [-4.93/17.45][a]	0.31	-10.70 [-19.72/-0.66] [a]	0.037	na	na
Ornithine, μmol/L	51.49 [34.48/70.66] [a]	<0.0001	7.6 [-4.57/21.32] [a]	0.23	na	na
ADMA, μmol/L	-19.20 [-26.70/-10.92][a]	<0.0001	-20.72 [-28.14/-12.54][a]	<0.0001	na	na
Arginine: Ornithine	1.88 [-5.87/10.27] [a]	0.65	6.49 [-1.68/15.34] [a]	0.12	na	na
GABR	13.54 [7.11/20.35] [a]	<0.0001	13.24 [6.77/20.10] [a]	<0.0001	na	na
Arginine:ADMA	91.38 [66.84/119.54] [a]	<0.0001	44.71 [26.01/66.17] [a]	<0.0001	na	na
**Endothelial function**	**Adj Coefficient [95% CI]**	**P-value**	**Adj Coefficient [95% CI]**	**P-value**	**Adj Coefficient [95% CI]**	**P-value**
FMD, %	1.19 [0.52 – 1.85][b]	<0.0001	0.93 [0.25 – 1.61][b]	0.008	1.04 [0.46 - 1.63]	<0.0001
Brachial artery diameter at rest, mm	0.02 [-0.02 /0.07][c]	0.3	0.09 [0.04 – 0.13][c]	<0.0001	0.06 [0.01 - 0.10]	0.007
Baseline blood flow [VTI]	0.021 [0.002 – 0.040][d]	0.029	0.039 [0.019 – 0.058][d]	<0.0001	0.030 [0.133 - 0.0468]	<0.0001
Reactive hyperemia absolute [RH]	0.03 [-0.01/0.07][e]	0.15	0.03 [-0.01/0.07][e]	0.12	0.03 [-0.004 - 0.066]	0.085
**Non-endothelial dependent vasodilation**						
GTN, %	-0.04 [-0.48/0.40][f]	0.86	0.08 [-0.37/0.54][f]	0.72	0.02 [-0.36 - 0.41]	0.911
**Haemoglobin**						
Hemoglobin, g/dL, mean [95% CI]	0.21 [0.09 – 0.32][g]	<0.0001	0.30 [0.19 – 0.42][g]	<0.0001	0.28 [0.19 - 0.37]	<0.0001
**C-reactive protein and hemolytic markers**	**Adj Coefficient (% change) [95% CI]**	**P-value**	**Adj Coefficient (% change) [95% CI]**	**P-value**	**Adj Coefficient (% change) [95% CI]**	**P-value**
C-reactive protein, mg/L	11.60 [-5.27/31.49][g]	0.19	14.58 [-2.89/35.21] [g]	0.11	13.08 [-1.30/29.58]	0.077
Lactate dehydrogenase, IU/L	7.70 [2.34 – 13.35]$_g$	0.004	5.17 [0.03 – 10.57] [g]	0.049	6.39 [2.21/10.73]	0.003
Total bilirubin, mg/dL	-14.42 [-21.86 - -6.26][g]	0.001	-6.52 [-14.71 – 2.45] [g]	0.15	-10.56 [-17.07/-3.52]	0.004
Non-conjugated bilirubin, mg/dL	-13.90 [-22.03 - -4.93][g]	0.003	-11.45 [-19.70 - -2.34] [g]	0.015	-12.68 [-19.53/-5.24]	0.001
**Nutritional status and body composition**					**Adj Coefficient [95% CI]**	**P-value**
Height for age z-score	na	na	na	na	0.021 [0.009 – 0.033][h]	0.001
Weight for age z-score	na	na	na	na	0.107 [0.070 -0.144][h]	<0.0001
Body mass index for age z-score	na	na	na	na	0.135 [0.081 – 0.188][h]	<0.0001
Whole body fat free mass (kg)	na	na	na	na	0.17 [0.08 -0.26][h]	<0.0001
Whole body fat (%)	na	na	na	na	0.53 [0.28 – 0.77][h]	<0.0001

a – adjusted for sex [0=female, 1=male] as level 2 variable, plus age [years], BMI-z-score and weight category [0=< 25kg, 1= ≥25kg] at each visit [i.e., level 1 variables].

b –Adjusted for sex as level 2 variable, plus age, weight category, resting brachial artery diameter, resting heart rate, RH & CRP as level 1 variables. Also, excludes one extreme outlier value at baseline for one subject.
c – adjusted for sex as level 2 variable , plus age, weight category, and resting diastolic blood pressure as level 1 variables.
d - adjusted for sex as level 2 variabl , plus age,weight category, resting brachial artery diameter, resting diastolic blood pressure & CRP at each visit as level 1 variables.
e - adjusted for sex as level 2 variable, plus age, weight category and resting heart rate as level 1 variables
f – adjusted for sex as level 2 variable, plus age, weight category, resting brachial artery diameter & CRP as level 1 variables.
g – adjusted for sex as level 2 variable, plus age and weight category as level 1 variables
h – Adjusted for sex as level 2 variable, plus age and seasonality as level 1 variables.* age^2 *was included as a random effect in models of whole body fat free mass and whole-body fat %*
** Season is a continuous variable ranging from 0 to 1 [where 0 is the 1st January and 1 is the 31st December] and was modelled as the first two sets of Fourier terms (first set =* $Cosine(2\pi S)$ *and* $Sine(2\pi S)$*; second set =* $Cosine(4\pi S)$ *and* $Sine(4\pi S)$*)*

Table S5. Number of reported painful episodes managed at home by intervention or washout period.

	Intervention	Washout
Reported number of times child unwell & managed at home [%]	245/2,555 [9.6]	203/3,174 [6.4]
Reported number of times child had pain managed at home [%]	128/2,555 [5.0]	92/3,174 [3.1]
Proportion of events managed at home with reported pain [%]	128/153 [83.7]<br>128/245 [52.2]*	92/142 [64.8]<br>92/203 [45.3]*

Participants families were interviewed alternatively at home and by telephone approximately every 7 days and asked if the participating child had been unwell since the last interview. If subject answered yes, then further questions were asked about the nature of the episode/s of being unwell.

**Proportion including missing values for pain variable [Y/N] in the denominator.*

Table S6. Laboratory adverse events by treatment period.

Laboratory marker	Severity	After washout	After any RUSF	After RUSF-v	After RUSF-b
**Elevated creatinine**	All grades	2/233 [0.9]	4/234 [1.7]	2/118 [1.7]	2/116 [1.7]
	Mild	1	2	1	1
	Moderate	0	1	1	0
	Severe	0	1	0	1
	V. Severe	1	0	0	0
**Elevated AST**	All grades	5/233 [2.1]	49/234 [21.0]	25/118 [21.2]	24/116 [20.1]
	Mild	5	47	23	24
	Moderate	0	1	1	0
	Severe	0	1	1	0
	V. Severe	0	0	0	0
**Elevated ALP**	All grades	0/233 [0]	11/234 [4.7]	6/118 [5.1]	5/116 [4.3]
	Mild	0	10	5	5
	Moderate	0	1	1	0
	Severe	0	0	0	0
	V. Severe	0	0	0	0
**Elevated Total Bilirubin**	All grades	6/233 [2.6]	10/234 [4.3]	5/118 [4.2]	5/116 [4.3]
	Mild	6	9	4	5
	Moderate	0	0	0	0
	Severe	0	1	1	0
	V. Severe	0	0	0	0
**Elevated WBC**	All grades	3/233 [1.3]	1/235 [0.4]	0/118 [0]	1/117 [0.9]
	Mild	2	0	0	0
	Moderate	0	0	0	0
	Severe	1	0	0	0
	V. Severe	0	1	0	1
**Low WBC**	All grades	2/233 [0.9]	4/235 [1.7]	1/118 [0.8]	3/117 [2.6]
	Mild	1	1	0	1
	Moderate	1	3	1	2
	Severe	0	0	0	0
	V. Severe	0	0	0	0
**Elevated Neutrophils**	All grades	4/232 [1.7]	8/235 [3.4]	3/118 [2.5]	5/117 [4.3]
	Mild	4	8	3	5
	Moderate	0	0	0	0
	Severe	0	0	0	0
	V. Severe	0	0	0	0
**Elevated lymphocytes**	All grades	0/232 [0]	0/235 [0]	0/118 [0]	0/117 [0]
**Elevated platelets**	Mild	2/232 [0.9]	2/235 [0.9]	2/118 [1.7]	0/117 [0]
	Moderate	0	0	0	0

	Severe	0	0	0	0
	V. Severe	0	0	0	0
**Low platelets**	All grades	2/233 [0.9]	5/235 [2.1]	2/118 [1.7]	3/117 [2.6]
	Mild	2	2	0	2
	Moderate	0	1	1	0
	Severe	0	0	0	0
	V. Severe	1	2	1	1
**Change in hemoglobin**	All grades	35/233 [15.0]	15/233 [6.4]	9/117 [7.7]	6/116 [5.2]
	Mild	31	14	9	5
	Moderate	2	0	0	0
	Severe	1	1	0	1
	V. Severe	1	0	0	0

REFERENCES

1. Little JA, Hauser KP, Martyr SE, et al. Hematologic, biochemical, and cardiopulmonary effects of L-arginine supplementation or phosphodiesterase 5 inhibition in patients with sickle cell disease who are on hydroxyurea therapy. *Eur J Haematol* 2009; **82**(4): 315-21.
2. Morris CR, Morris SM, Jr., Hagar W, et al. Arginine therapy: a new treatment for pulmonary hypertension in sickle cell disease? *Am J Respir Crit Care Med* 2003; **168**(1): 63-9.
3. Waugh WH, Daeschner CW, 3rd, Files BA, McConnell ME, Strandjord SE. Oral citrulline as arginine precursor may be beneficial in sickle cell disease: early phase two results. *J Natl Med Assoc* 2001; **93**(10): 363-71.
4. Schwedhelm E, Maas R, Freese R, et al. Pharmacokinetic and pharmacodynamic properties of oral L-citrulline and L-arginine: impact on nitric oxide metabolism. *Br J Clin Pharmacol* 2008; **65**(1): 51-9.
5. Donald AE, Halcox JP, Charakida M, et al. Methodological approaches to optimize reproducibility and power in clinical studies of flow-mediated dilation. *J Am Coll Cardiol* 2008; **51**(20): 1959-64.
6. Charakida M, Donald AE, Green H, et al. Early structural and functional changes of the vasculature in HIV-infected children: impact of disease and antiretroviral therapy. *Circulation* 2005; **112**(1): 103-9.
